# Prospective 5-year study with 96 short curved Fitmore™ hip stems shows a high incidence of cortical hypertrophy with no clinical relevance

**DOI:** 10.1186/s13018-019-1174-1

**Published:** 2019-05-27

**Authors:** Caroline Thalmann, Patricia Kempter, Karl Stoffel, Thea Ziswiler, Arno Frigg

**Affiliations:** 1 Orthopedic Department, Kantonsspital Graubünden, Loestrasse 99, 7000 Chur, Switzerland; 2 Orthopedic Department, University Hospital Basel, Spitalstrasse 21, 4031 Basel, Switzerland; 3 Orthopedic Department, Kantonsspital Liestal, Rheinstrasse 26, 4410 Liestal, Switzerland; 4 Private University of the Principality of Liechtenstein, Triesen, Liechtenstein

**Keywords:** Cortical hypertrophy, Long term, Short stem, Fitmore, Clinical relevance

## Abstract

**Background:**

An increased occurrence of cortical hypertrophy (CH) was observed 1–2 years after implanting short curved Fitmore hip stems. There are no published data about either the clinical relevance or the progression of CH over the long term.

**Methods:**

Ninety-six primary total hip arthroplasties were performed between 2008 and 2010 using the Fitmore hip stem. Clinical and radiological parameters were recorded preoperatively and at 1, 2, 3, and 5 year follow-up.

**Results:**

CH appeared mainly on antero-posterior radiographs in Gruen Zones 2, 3, 5, and 6. After 1 year, the diameter was 10 ± 2 mm and remained constant thereafter. The CH rate after 1 year was 69% and after 5 years 71%. Subsidence after 1 year was 1.6 ± 1.55 mm and 1.93 ± 1.72 mm after 5 years. Cortical thinning was 46% after 1 year and 56% after 5 years, mainly in Gruen Zones 7 and 8. In the first year radiolucencies were found in 51% in all Gruen Zones, and in 20% after 5 years. Patient, implant, and surgical factors did not correlate with radiological outcomes except that larger stems had more CH. After 5 years, the Harris Hip Score had improved from 59 to 94 and the Oxford Hip Score from 22 to 41. Radiographic parameters, notably CH, were not associated with clinical outcomes except that cortical thinning correlated with lower outcome scores.

**Conclusions:**

CH correlated neither with clinical outcome nor with patient, surgical or implant factors, except for a positive correlation with stem size. The Fitmore hip stems settled within the first year to a stable fixation and then remained almost unchanged. However, cortical thinning is common in Gruen Zone 7 and 8 meaning that there is stress-shielding.

## Background

Cortical hypertrophy (CH) is one of several observed bone remodeling mechanisms after total hip arthroplasty (THA). It is a thickening of cortical bone in an adaptive response to altered external mechanical loads, which can cause internal stress and proximal cortical thinning in the femur [1, 2]. Initially, CH was observed around cemented stems [2]. The intensified appearance around uncemented stems is explained by stress-shielding, a reactive proximal bone atrophy combined with distal bone hypertrophy caused by aberrant loading through distal site [3, 4]. The increased remodeling appears to be due to greater bending stiffness, which depends on stem material and cross-sectional size and shape [5, 6]. By improving the stem design, the incidence of CH in conventional long straight stems could be reduced [7–9].

To reduce proximal stress-shielding and to facilitate minimally invasive approaches, short curved stems with proximal fixation were developed [10, 11]. These stems are easier to implant through small incisions than conventional long straight stems [7, 10, 11]. Short stems are thought to facilitate future revision due to decreased proximal bone hypotrophy, although this is not supported by evidence [10]. To preserve more bone stock, the new short stems must bear load more proximally. To achieve this, major changes in femoral stem design were necessary that carried the risk of possible upcoming adverse effects like distal CH [12, 13].

Follow-up data after 1–2 years are available for the new short curved Fitmore hip stem [10, 16]. Biomechanical tests showed the new stem to have a lower stress-shielding leading to increased physiological cortical strain, lower micromotion, and reduced migration compared to the conventional stem [14, 15]. However, 1–3 years after implantation, radiographs revealed CH around the distal part of the Fitmore hip stem (Gruen Zones 3 and 5) in 29–63% [10, 16, 17].

The significance of CH remained unclear. In the retrospective short term (1–3 years), no clinical difference was found between patients with or without CH [10, 16]. The results may have started a competition between the new short stems and the conventional straight stems that have provided good results for three decades [18, 19]. This study aims to evaluate for the first time prospectively in a cohort with a follow-up of 5 years the clinical relevance of CH after implantation of the short curved Fitmore hip stem and to assess the effect of patient, surgical, and implant factors on the occurrence of CH.

## Methods

This prospective study was approved by the local Ethical Review Board and all patients have provided written informed consent. The study was carried out in accordance with the declaration of Helsinki and the applicable laws.

**Fig. 1:**
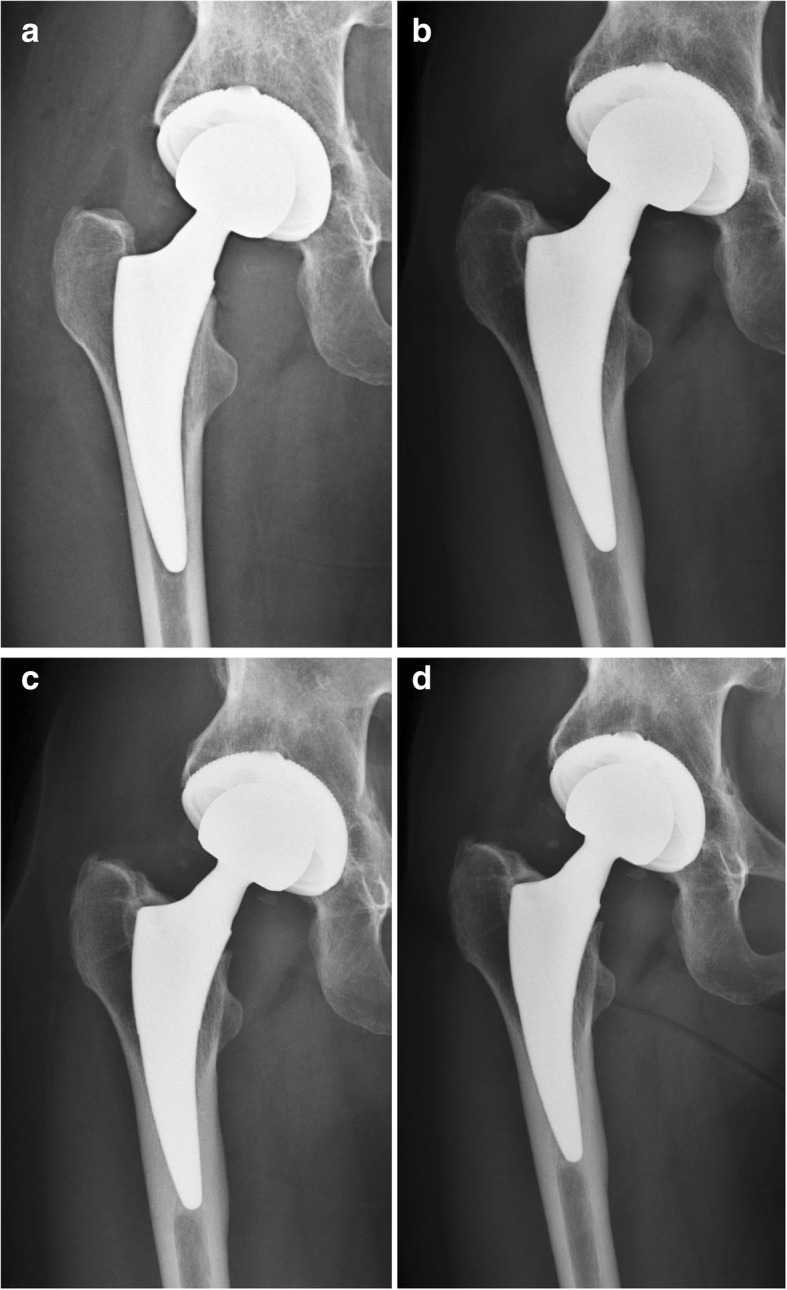
Radiographs of a 50-year-old female patient with a typical course of a short femoral Fitmore hip stem. **a** immediately after surgery with radiolucencies at the lesser trochanter, **b** at 1 year postoperatively showing cortical hypertrophy in Gruen zones 3, 5 and 6, and a decrease in radiolucencies, but also cortical thinning around the lesser trochanter, **c** at 2 years with equal findings, **d** after 5 years with cortical hypertrophy, disappearance of radiolucencies, but with cortical thinning at the level of the lesser trochanter

From April 2008 to April 2010 a total of 123 of primary THA were performed in 120 consecutive patients using the Fitmore hip stem (Zimmer Biomet, Winterthur, Switzerland, Fig. 1), a cementless, curved, solid short stem with a trapezoidal cross-section and metaphyseal anchoring. Osteointegration is achieved through a proximal Porolock® titanium coating. Inclusion criteria were the implantation of the Fitmore hip stem in the time frame of the study and the availability of complete clinical and radiological data at all follow-up visits up to 5 years. Exclusion criteria were lost to follow-up (14), death (6 patients with 7 operated hips), missing data (5), and revised hips (1). Thus, a total of 96 Fitmore hip stems (95 patients) could be evaluated. Mean patient age was 62 ± 10 years; 40% were of female gender and 60% male. The Dorr classifications were 82% A, 18% B, and 0% C. The indications for THA were osteoarthritis (92%), acute fracture (3%), osteonecrosis (2%), post-traumatic arthritis (2%), and hip dysplasia (1%, Table 1).

**Table 1:** Patient, implant, and surgical data

Patient demographics	
*N*	96
Operated side (R/L)	54%/46%
Mean age at time of operation (SD)	62.32 (± 9.97)
Gender (M/F)	60.4%/39.6%
Dorr Index (A/B/C)	82.3%/17.7%/0%
Fitmore hip stem family (A/B/B extended/C)	9.4%/47.9%/40.6%/2.1%
Leg axis [°] (Varus/Valgus)	94.1%/5.9%
Median stem size (range)	7 (5–8)
Mean preoperative leg-length difference [cm] (SD)	− 2.75 (± 5.08)
Mean postoperative leg-length difference [cm] (SD)	− 0.8 (± 6.18)
Mean difference between preoperative and postoperative leg-length difference [cm] (SD)	4.13 (± 3.57)
Mean preoperative offset [mm] (SD)	38.24 (± 7.73)
Mean postoperative offset [mm] (SD)	40.4 (± 7.43)
Mean difference between preoperative and postoperative offset [mm] (SD)	2.16 (± 7.17)
Varus/valgus position after surgery	36.5%/0%/63.5% neutral

### Surgery and postoperative protocol

The Fitmore hip stems were implanted in a large general hospital by various orthopedic surgeons as standard implant in all consecutive cases with no contraindications except decreased bone quality (Dorr type C) on the preoperative radiographs. In these cases, a cemented stem was used. The approach was anterolateral, minimally invasive in 80%, and standard lateral in 20% of cases. A Fitmore cup was used in 90% and a trabecular metal modular cup was used in 10% in case of decreased bone quality or press-fit of the Fitmore cup (Zimmer Biomet, Winterthur, Switzerland) and a CoCr head. In the time period of this study, two senior surgeons at our institution generally declined minimally invasive surgery and implanted other uncemented hip stems (straight CLS stem, the standard before minimally invasive surgery) using an open approach. All patients started full weight-bearing activities with a 4-point crutch gait immediately after the operation.

### Prospective evaluation

Patients were clinically documented at baseline, immediately postoperatively, and followed-up after 1, 2, 3 and 5 years with a physical examination, documentation of thigh pain, EQ-5D, Harris Hip Score (HHS), and Oxford Hip Score. Antero-posterior (AP) and axial radiographs of both hips were taken with internally rotated legs. Standardization was achieved by placing a 25-mm radiopaque gage ball between the thighs of the patient (at baseline) and by using the normalized implant head diameter according to type size (for postoperative radiographs).

### Radiological evaluation

All measurements were performed using the MediCAD (Hectec, Germany) operation planning program. Parameters were assessed using the PACS-Web-Viewer program. The following parameters were measured (Table 2) [13, 20–23]: (1) *cortical hypertrophy*: the extent of CH was evaluated through measuring the maximal distance from the inner to the outer edge of the cortical bone at a right angle to the stem axis; (2) *bone condensation*: bone condensation describes the reaction of cancellous bone to stem implantation; as in cortical hypertrophy, a radiologically denser area can be observed, usually located below the tip of the stem; (3) *cortical thinning*; (4) *radiolucency*; (5) *reactive lines*; (6) *osteolysis*; (7) *calcar rounding*; (8) *calcar resorption*; (9) *subsidence*: the difference between the level of the shoulder of the implant and a parallel line of the trochanteric uppermost tip in AP radiograph between the immediate postoperative and later follow-up radiographs), and (10) *varus/valgus position*: the angle between the axis of the stem defined as the most distal point of the stem and the midway point between stem shoulder and outer stem neck and the femur. The neutral position was defined as 0 ± 5°, higher positive values as varus and higher negative values as valgus.

**Table 2:** Radiological parameters after 5 years

Radiographical parameter	
CH	71%
Bone condensation	98%
Cortical thinning	76%
Radiolucency	22%
Reactive lines	17%
Osteolysis	75%
Calcar rounding	85%
Calcar resorption	3%
Mean subsidence [mm] (SD)	− 1.93 (± 1.72)

### Statistics

Patient parameters (age, gender, Dorr Index), implant parameters (stem type, stem size), surgical parameters (leg length difference, offset, varus/valgus), radiological parameters (Table 2), and clinical outcome parameters (Table 3) were analyzed descriptively. Mean and standard deviation are reported. The correlation between radiological and clinical outcomes was analyzed. Patient, implant, and surgical parameters were analyzed for correlation with radiological and clinical outcome parameters. Binary outcome parameters assessed in AP and axial radiographs were collapsed into one single variable: “yes” in either AP or axial was coded as “yes,” and “no” in both AP and axial was recoded as “no.” Categorical variables were compared with a chi-square test, and continuous variables were compared with Wilcoxon rank sum test (for two groups) or Kruskal-Wallis test (for more than two groups). Kendall’s rank correlation was computed for two ordinal variables. All analyses were performed in the R programming language (version 3.3.3) [24]. The package “tableone” [25] was used for description of baseline and clinical characteristics. No correction for multiple tests was performed in this explorative study.

**Table 3:** CH and Subsidence: appearance in Gruen Zones over course of 5 years

	1 year	2 year	3 year	5 year
Cortical hypertrophy				
AP: Y/N	68.8%	68.8%	69.8%	70.8%
AP Gruen zone 1	–	–	–	–
AP Gruen zone 2	42%	40%	42%	40%
AP Gruen zone 3	58%	57%	57%	59%
AP Gruen zone 4	–	–	–	–
AP Gruen zone 5	43%	44%	45%	46%
AP Gruen zone 6	16%	18%	18%	18%
AP Gruen zone 7	–	–	–	–
Mean medial distance [mm] (SD)	10.42 (± 2.08)	10.53 (± 2.09)	10.48 (± 2.19)	10.38 (± 2.14)
Mean lateral distance [mm] (SD)	9.89 (± 2.05)	9.92 (± 2.09)	9.75 (± 2.01)	9.72 (± 2.04)
Axial: Y/N	11.5%	11.5%	11.5%	11.5%
Axial Gruen zone 8	2%	2%	2%	2%
Axial Gruen zone 9	6%	5%	5%	5%
Axial Gruen zone 10	2%	3%	3%	3%
Axial Gruen zone 11	–	–	–	–
Axial Gruen zone 12	5%	6%	5%	6%
Axial Gruen zone 13	3%	2%	3%	3%
Axial Gruen zone 14	–	–	–	–
Mean ventral distance [mm] (SD)	9.09 (± 2.51)	9.9 (± 2.69)	9.4 (± 2.37)	9.55 (± 2.58)
Mean dorsal distance [mm] (SD)	9.45 (± 3.53)	9 (± 3.5)	3.5 (± 3.24)	9.55 (± 3.42)
Subsidence				
[mm] (SD)	− 1.62 (± 1.55)	− 1.78 (± 1.6)	−1.8 (± 1.67)	−1.93 (± 1.72)

From the large amount of radiological data collected during 5 years of follow-up, we report only on CH, cortical thinning, and radiolucency because they are the most important parameters and because cortical thinning represents to some extent calcar rounding and osteolysis.

## Results

### Radiographic results

The radiographic results are summarized in Table 2. CH occurred mainly on the AP radiographs in Gruen Zones 2, 3, 5, and 6 (Table 3, Fig. 1). After 1 year, the maximal diameter of CH on the medial cortex was 10.4 ± 2 mm and on the lateral cortex 9.9 ± 2 mm, which both remained constant over the course of 5 years. The rate of CH after 1 year was 69%, increasing slightly to 71% over 5 years (Table 3).

Cortical thinning was 46% after 1 year, increasing to 56% after 5 years. In the AP view, cortical thinning predominantly occurred in Gruen Zone 7 with 43%, but it was also detectable in Gruen Zones 1, 2, 6 (Table 4, Fig. 1). Cortical thinning increased over 5 years in all Gruen Zones (e.g., to 51% in Gruen Zone 7). In the axial view, cortical thinning occurred mainly in Gruen Zone 8 with 38%, but also in Gruen Zones 9 and 12–14, increasing in all zones over 5 years (e.g., to 51% in Gruen Zone 8).

**Table 4:** Cortical Thinning: appearance in Gruen Zones over the course of 5 years

Cortical thinning	1 year	2 year	3 year	5 year
AP: Y/N	46%	52%	55%	56%
AP Gruen zone 1	2%	5%	7%	7%
AP Gruen zone 2	3%	5%	9%	11%
AP Gruen zone 3	–	–	–	–
AP Gruen zone 4	–	–	–	–
AP Gruen zone 5	–	–	–	–
AP Gruen zone 6	9%	14%	16%	17%
AP Gruen zone 7	43%	48%	51%	51%
Axial: Y/N	46%	59%	63%	70%
Axial Gruen zone 8	38%	45%	43%	51%
Axial Gruen zone 9	14%	24%	24%	27%
Axial Gruen zone 10	–	–	5%	4%
Axial Gruen zone 11	–	–	–	–
Axial Gruen zone 12	4%	8%	8%	13%
Axial Gruen zone 13	10%	18%	22%	23%
Axial Gruen zone 14	2%	5%	5%	7%

Radiolucencies occurred in the first year in 51% on the AP and axial radiographs in all Gruen Zones except Zone 2, diminishing to 20% over 5 years (Table 5, Fig. 1).

**Table 5:** Radiolucencies: appearance in Gruen Zones over the course of 5 years

Radiolucencies	1 year	2 year	3 year	5 year
AP: Y/N	51%	38%	28%	20%
AP Gruen zone 1	41%	31%	26%	19%
AP Gruen zone 2	–	2%	2%	–
AP Gruen zone 3	13%	7%	2%	–
AP Gruen zone 4	17%	9%	4%	–
AP Gruen zone 5	16%	8%	3%	–
AP Gruen zone 6	10%	3%	3%	2%
AP Gruen zone 7	13%	7%	55	2%
Axial: Y/N	35%	21%	17%	9%
Axial Gruen zone 8	26%	16%	14%	9%
Axial Gruen zone 9	4%	5%	3%	2%
Axial Gruen zone 10	11%	8%	6%	–
Axial Gruen zone 11	9%	6%	5%	–
Axial Gruen zone 12	11%	8%	5%	–
Axial Gruen zone 13	5%	6%	4%	3%
Axial Gruen zone 14	13%	7%	6%	6%

Subsidence affected 77% after 1 year and this rate remained constant thereafter. Subsidence was 1.6 ± 1.55 mm after 1 year and increased gradually to 1.93 ± 1.72 mm after 5 years (Table 3). Bone condensation (98%), cortical thinning (76%), osteolysis (75%), and calcar rounding (85%) were frequent (Table 2).

### Clinical results

After 5 years, the Harris Hip Score improved from 59.2 ± 15.6 to 93.8 ± 10.25 and the Oxford Hip Score improved from 22.2 ± 8.5 to 41.02 ± 9.07. Patient satisfaction was 99% (Table 6). There were no implant failures and survival was 99%: 1 of 102 Fitmore stems showed an aseptic loosening after 18 months that was treated by removing the implant and replacing it with a straight uncemented CLS stem. Complications included 1 hip dislocation (after mobilization on the first postoperative day; treated with closed reposition; it resolved thereafter), 1 postoperative hematoma (on the 11th postoperative day; it resolved with conservative therapy), and 1 wound infection (deep infection after 7 days, treated with open debridement, irrigation, and antibiotics; it resolved completely with the implant in place). Most patients experienced no (84%) or only slight (13%) thigh pain. Similarly, most patients experienced no (72%) or at most moderate pain (27%) on the EQ5D-severity scale.

**Table 6:** Descriptive statistics of clinical parameters

Clinical parameter	
Patient satisfaction (Y/N)	98.8%/1.2%
Thigh pain (none/slight/moderate/severe)	84.2%/12.6%/2.1%/1.1%
EQ5D-severity of pain (none/moderate/excessive)	71.6%/27.3%/1.1%
Mean EQ5D score (SD)	0.9 (± 0.16)
Mean Harris Hip Score (SD)	93.8 (± 10.25)
Oxford Hip Score	41.02 (9.07)
Oxford-severity of pain (none/very mild/mild/moderate/severe)	56.7%/14.4%/14.4%/5.6%/8.9%

### Effect of patient, implant, or surgical factors on radiological outcome

Patient parameters did not correlate with radiological outcomes, except for cortical thinning which was more frequent in females (87%) than in males (69%, *P* = 0.045). Implant parameters did not correlate with radiological outcomes, except that CH was more frequent in stem sizes 9–12 (94.1%) than in stem sizes 5–8 (66.2%) or sizes 1–4 (64.3%; *P* = 0.05).

Surgical parameters did not correlate with radiological outcomes. Subsidence was significantly larger when CH (median − 2 mm, IQR − 3 to − 1; *P* = 0.013) was present than when it was absent (median − 1 mm; IQR − 2 to 0).

### Effect of radiological parameters on clinical outcome

Radiographic parameters did not correlate with clinical outcome or pain, except for cortical thinning which showed a lower EQ5D-score (median 1, IQR 0.8–1 versus 1, 1–1; *P* = 0.014), a lower Harris Hip Score (median 96, IQR 21–100 versus 100, IQR 97–100; *P* = 0.01) and a lower Oxford hip score (median 45, IQR 37–48 versus 48, IQR 42–48; *P* = 0.033), compared to patients without cortical thinning. However, these differences had no clinical significance or consequences. There was no difference in the clinical scores or pain subscores in patients with or without CH (EQ5D *P* = 0.988, Harris Hip Score *P* = 0.975, Oxford Severity of Pain *P* = 0.172).

## Discussion

This is the first prospective study analyzing clinical and radiographical parameters of a cohort of 96 Fitmore hip stems over 5 years to evaluate the rate and clinical relevance of CH and factors affecting CH. Follow-up data beyond 3 years have not been published so far [10, 16].

### CH in short femoral stems

We found 71% of CH after 5 years. The literature reports 29–63% after 1–3 years for the Fitmore hip stem [10, 16]. No 5-year data are available. In contrast to the Optimys stem, another short curved femoral stem, only 4.4% of CH were found after 2 years [26]. This difference can be explained by the large proximal taper of the Optimys stem, which leads to higher primary rotation stability and increased proximal stress loading. Also, the polished tip of the Optimys stem prevents bone ingrowth and load transfer and, as a calcar guided stem, usually has no bone contact. In contrast, the Fitmore stem has a 3-point fixation allowing for bone contact at the tip, thereby causing CH.

### CH in straight stems

In straight stems, CH has been found to occur in 2–10% [3, 27–30], which is much lower than in this study with the Fitmore hip stem. One reason may be that conventional straight stems better follow the bending of the bone whereas the shorter Fitmore stem acts more rigidly [15]. Another reason may be that the Fitmore hip stem primarily has three points of bone contact (calcar proximally Gruen Zone 7, lateral endostal surface Gruen Zone 2 and 3, medial tip Gruen Zone 5). After settling down with some subsidence, it has a 2-point bone contact distally with increased load transfer, whereas straight stems have a larger total bone contact surface. Therefore, the Fitmore stems have a different distribution of CH than straight stems which also show CH in Gruen Zone 6 in addition to 2, 3 and 5 [3, 29, 30].

### Progression of CH over 5 years

This study is the first to report on the Fitmore hip stem’s settling over time. We found that CH and subsidence remained constant over the course of 5 years and that the Fitmore hip stem subsides within the first year and changes minimally thereafter. This is in line with other stem types, where an initial remodeling is observed in the first 6–12 months with almost no further changes after 3–4 years [23, 31].

### Clinical relevance

We found that CH affects neither the clinical outcome nor thigh pain, which is in line with other findings [27, 29, 31–33].

### Subsidence

We found a subsidence of 1.6 mm (± 1.6) after 1 year and 1.93 mm (± 1.7) after 5 years. This is in accordance with other studies with short and long stems which reported a subsidence of 1–4 mm [34–36]. In the literature, a subsidence of less than 2–5 mm is accepted as normal [34–36]. Our subsidence of 1–2 mm could be interpreted as micromotion and as a sign of a stable implant [10, 12, 18, 23, 35–38]. Subsidence was significantly higher (≤ 2 mm) in the presence of CH than in its absence (≤ 1 mm). Our findings contrast with Cho et al. [29] who report subsidence to have been lower in hips with CH compared to hips without CH (1.5 versus 3.4 mm). We explain the increased CH in case of subsidence with the primary stability of the Fitmore stem: in case of an optimal press-fit after reaming, the stem has a 3-point fixation (proximal calcar, lateral cortex, medial tip) while if the press-fit was not optimal, the Fitmore stem subsides and locks distally with a 2-point load transfer causing CH.

### Stress-shielding

Stress-shielding of the proximal femur has been observed in a number of conventional cementless implants used in THA [5, 30, 39, 40]. Short femoral-neck implants were originally invented to reduce interference with the biomechanics of the proximal femur [12, 41]. In theory, the Fitmore stem was designed to have a 3-point fixation to avoid proximal bone loss. However, our study shows that in reality, the Fitmore stem exhibits a distal 2-point fixation with distal load transfer and proximal stress-shielding. This explains our finding of a high rate of cortical thinning in 56% of cases after 5 years, which is in line with the literature on other short stems. With the short Mayo stem, bone mineral density had decreased significantly 2 years after surgery [12, 38, 42]. For Nanos and Mayo short stems, a bone loss of 30–69% in Gruen zones 1 and 7 was also reported [42, 43]. In summary, a substantial loss in the proximal periprosthetic bone cannot be prevented by using short curved stems because forces are distally transmitted [12]. We found lower EQ5D, Harris Hip, and Oxford Hip scores with cortical thinning. Although these differences were statistically significant, the absolute differences were small and clinically not significant after 5 years. Future research is needed to evaluate potential consequence in the long-term.

### Clinical outcome

Maier et al. reported an HHS of 94, which equals our results [10]. We found 3.3% of moderate to severe thigh pain, which is also in agreement with the results of Maier et al. who reported 4% of thigh or hip pain [10]. Cinotti, using a short femoral stem, found thigh pain in 8% of patients after 2 years, which reduced to 3% after 9 years [34].

### Survival

Our survival rate of 99% at 5 years and the 100% reported by Maier et al. [10] after 2 years confirm the encouraging results for the Fitmore hip stem. Patel et al. using a short custom stem found 100% survival at 5 years [44]. However, long-term studies with 10–20 years of follow-up are needed to establish whether the short stems can challenge the conventional stems.

### Limitations

A study with novel THA-implants requires a 10-year follow-up to be conclusive. Our prospective study is still running and 10-year follow-up results will be available in a few years. Second, we have accumulated a large number of radiological parameters (Table 2) of which progression could be reported over 5 years. Because of space constraints, we have focused on the parameters reported in Tables 3, 4, and 5. The strengths of this study are the prospective design since all other publications are retrospective and that it reports with 5 years the longest follow-up [10, 16].

## Conclusions

This is the first prospective cohort study with 96 Fitmore hip stem THA with a 5-year follow-up. We could show that CH is frequent with Fitmore hip stems but did not correlate with clinical outcome and was not influenced by factors related to patient, implant, or surgical procedure. CH occurred within the first year and then remained constant. This means that the Fitmore hip stem settles within the first year to a stable fixation and then remains almost unchanged, with constant subsidence, and with radiolucencies even disappearing over the years. However, cortical thinning was frequent in Gruen Zones 7 and 8 meaning that there is stress-shielding.

## Abbreviations

AP: Antero-posterior
CH: Cortical hypertrophy
HHS: Harris Hip Score
IQR: Interquartile range
PACS: Picture archiving system
SD: Standard deviation
THA: Total hip arthroplasty
